# Anaerobic detoxification of acetic acid in a thermophilic ethanologen

**DOI:** 10.1186/s13068-015-0257-4

**Published:** 2015-05-09

**Authors:** A Joe Shaw, Bethany B Miller, Stephen R Rogers, William R Kenealy, Alex Meola, Ashwini Bhandiwad, W Ryan Sillers, Indraneel Shikhare, David A Hogsett, Christopher D Herring

**Affiliations:** Mascoma Corporation, Lebanon, NH 03766 USA; Novogy Inc., 85 Bolton St, Cambridge, MA 02140 USA; Verdezyne Inc., 2715 Loker Avenue West, Carlsbad, CA 92010 USA; Thayer School of Engineering, Dartmouth College, Hanover, NH 03755 USA; Energy Biosciences Institute, 2151 Berkeley Way, Berkeley, CA 94704 USA; Myriant Corporation, 66 Cummings Park, Woburn, MA 01801 USA; OPX Biotechnologies Inc., 2425 55th Street, Boulder, CO 80301 USA

**Keywords:** Acetate detoxification, Cellulosic ethanol, Metabolic engineering, Inhibitors

## Abstract

**Background:**

The liberation of acetate from hemicellulose negatively impacts fermentations of cellulosic biomass, limiting the concentrations of substrate that can be effectively processed. Solvent-producing bacteria have the capacity to convert acetate to the less toxic product acetone, but to the best of our knowledge, this trait has not been transferred to an organism that produces ethanol at high yield.

**Results:**

We have engineered a five-step metabolic pathway to convert acetic acid to acetone in the thermophilic anaerobe *Thermoanaerobacterium saccharolyticum*. The first steps of the pathway, a reversible conversion of acetate to acetyl-CoA, are catalyzed by the native *T. saccharolyticum* enzymes acetate kinase and phosphotransacetylase. *ack* and *pta* normally divert 30% of catabolic carbon flux to acetic acid; however, their re-introduction in evolved ethanologen strains resulted in virtually no acetic acid production. Conversion between acetic acid and acetyl-CoA remained active, as evidenced by rapid ^13^C label transfer from exogenous acetate to ethanol. Genomic re-sequencing of six independently evolved ethanologen strains showed convergent mutations in the *hfs* hydrogenase gene cluster, which when transferred to wildtype *T. saccharolyticum* conferred a low acid production phenotype. Thus, the mutated *hfs* genes effectively separate acetic acid production and consumption from central metabolism, despite their intersecting at the common intermediate acetyl-CoA. To drive acetic acid conversion to a less inhibitory product, the enzymes thiolase, acetoacetate:acetate CoA-transferase, and acetoacetate decarboxylase were assembled in *T. saccharolyticum* with genes from thermophilic donor organisms that do not natively produce acetone. The resultant strain converted acetic acid to acetone and ethanol while maintaining a metabolic yield of 0.50 g ethanol per gram carbohydrate.

**Conclusions:**

Conversion of acetic acid to acetone results in improved ethanol productivity and titer and is an attractive low-cost solution to acetic acid inhibition.

**Electronic supplementary material:**

The online version of this article (doi:10.1186/s13068-015-0257-4) contains supplementary material, which is available to authorized users.

## Background

Biological conversion of low-cost carbohydrate-based renewable resources [1] is often hindered by inhibitors present in these heterogeneous materials. Acetic acid has consistently been reported as one of the most significant inhibitors, followed by furfural, hydroxymethylfurfural, and phenolic compounds [2,3]. Unlike other inhibitors, which are by-products of specific chemical and physical pretreatment processes [4,5], acetic acid can result directly from acetylated carbohydrates, and its production is unavoidable during carbohydrate fermentation.

Acetic acid is a particularly potent inhibitor at pH values under 6, a range common for industrial fermentation [6-8]. Undissociated acetic acid (pKa 4.75) freely passes through cellular membranes, where it can then reach a new equilibrium with dissociated acetic acid at the intracellular pH. The net result is both uncoupling of the transmembrane pH gradient and an accumulation of acetate anion in the cytoplasm [9,10].

Several chemical and physical methods to separate or detoxify acetic acid prior to fermentation have been proposed [11], but they require additional expense to implement, which may hinder commercial application. Biological removal of acetate has also been proposed via aerobic respiration of acetate followed by fermentation of carbohydrates to ethanol [12,13]. However, this requires a separate oxygen-dependent incubation, which raises overall process costs. Solventogenic clostridia, typified by *Clostridium acetobutylicum*, have the native ability to convert acetic acid and butyric acid into the solvents acetone and butanol [14]. The conversion is not easily transferrable to the context of ethanol production though, as it normally occurs in two phases with acetate first produced in an acidogenic phase, followed by conversion to acetone in a solventogenic phase [15].

Acetate detoxification can occur under normal fermentation conditions, enabled by an advanced organism without requiring additional capital equipment. We report conversion of acetic acid to acetone while maintaining a high yield of carbohydrates to ethanol in *Thermoanaerobacterium saccharolyticum*, thereby demonstrating a low-cost solution to acetic acid inhibition.

## Results

### Re-introduction of *ack* and *pta* do not restore acetate production in evolved ethanologen strains

**Figure 1:**
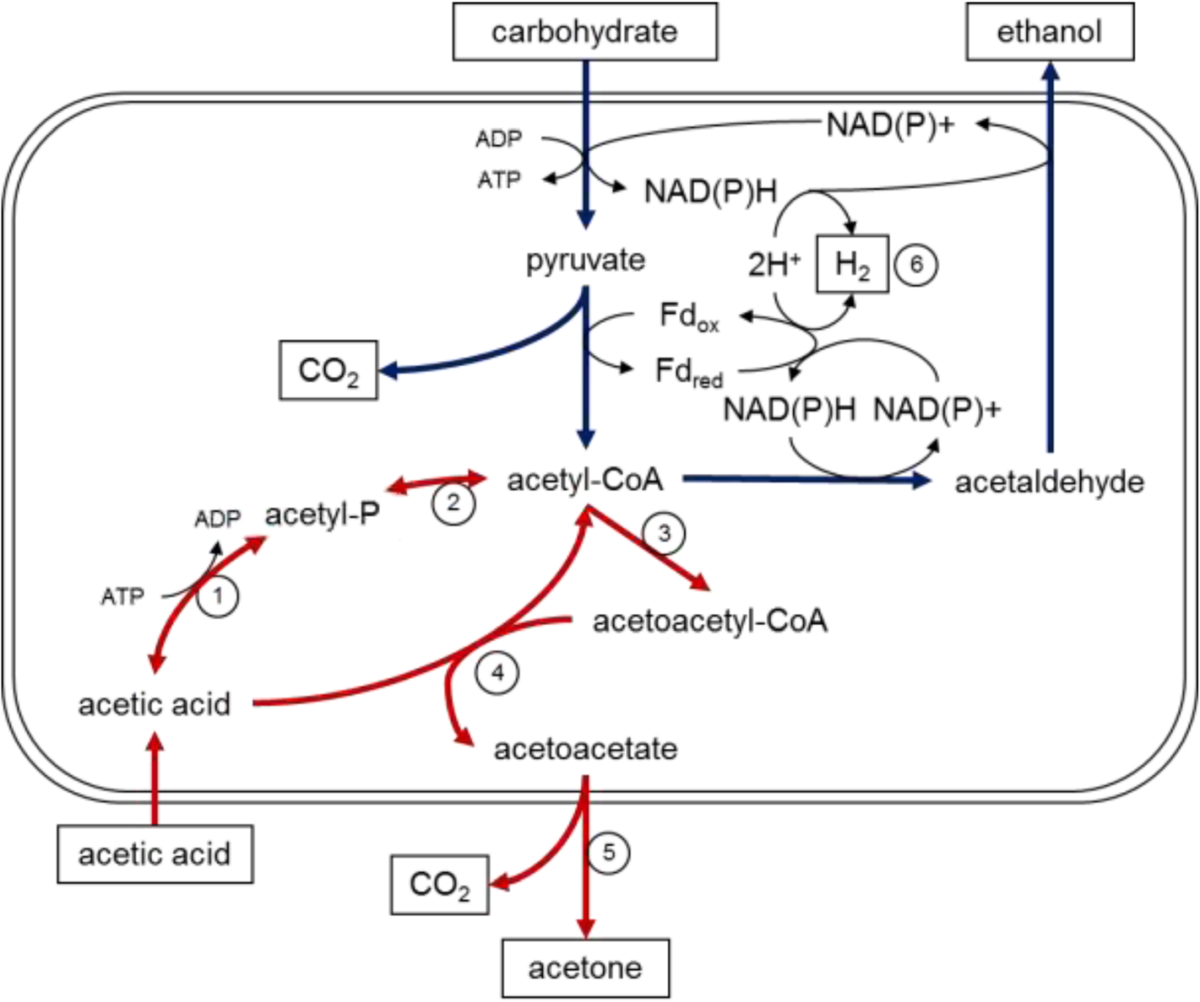
Synthetic pathway to convert acetic acid to acetone. Acetic acid diffuses freely into the cell following hydrolysis of acetylated polysaccharides and is then activated to acetyl-CoA by acetate kinase (1), phosphotransacetylase (2), and a half-reaction of CoA-transferase (4). Two acetyl-CoA molecules are then converted to acetoacetyl-CoA by thiolase (3), acetoacetate by the other half-reaction of CoA-transferase, and finally to acetone and CO_2_ by acetoacetate decarboxylase (5). Although the synthetic pathway shares a common intermediate with the ethanol production pathway, carbohydrate to ethanol production remains highly coupled due to the requirement to balance NAD(P)+/NAD(P)H generation. Hydrogenases (6) act to uncouple electron acceptor regeneration and ethanol formation, resulting in production of acetic acid through the reversible acetate kinase and phosphotransacetylase pathway.

**Figure 2:**
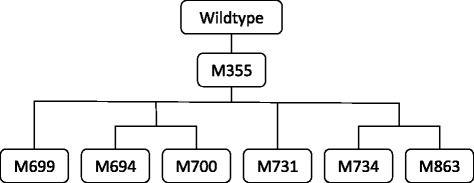
Lineage of strains selected for improved growth rate and ethanol production in this study. M0355 carries deletions of the *ldh*, *pta*, and *ack* but was not selected for improved growth rate. The lineages resulting in M0694, M0699, and M0700 were cultivated in continuous culture to select for improved growth properties. The lineages resulting in M0731, M0734, and M0863 were exposed to chemical mutagenesis and serial transfer for improved growth properties; the lineage resulting in M0863 was subsequently cultured in continuous culture. All strains were isolated as single colonies prior to genomic DNA isolation and re-sequencing.

*T. saccharolyticum* has previously been engineered to produce ethanol at high yield by deletion of *ldh* encoding L-lactate dehydrogenase and *ack* and *pta* encoding acetate kinase and phosphotransacetylase [16,17]. From a carbon-based perspective, these two deletions eliminate organic acid production and force central metabolic flux towards ethanol. From an electron-based perspective, nicotinamide adenine dinucleotide (NADH) generated during glycolysis and reduced ferredoxin generated during pyruvate cleavage must be re-oxidized through reduction of acetyl-CoA to ethanol, rather than through formation of lactic acid or molecular hydrogen (Figure 1). In attempts to engineer ethanologen strains with improved properties for industrial fermentation, we developed multiple strain lineages derived from strain M0355, a Δ*ldh* Δ*ack* Δ*pta* mutant [16]. Several of these lineages underwent evolutionary selection, either via continuous culture or serial transfer, for improved fermentation fitness (Figure 2). Interestingly, we found on several occasions that re-introduction of phosphotransacetylase and acetate kinase into evolved strains resulted in greatly reduced acetic acid production as compared to the same genes re-introduced into strain M0355 or the wildtype strain.

### Spontaneous mutations occurring during evolutionary selection direct electron flux to ethanol

One possible explanation for this phenomenon was that mutations which occurred during the evolutionary process direct metabolic flux to ethanol even in the presence of a functional pathway from acetyl-CoA to acetic acid. We re-sequenced the genomes of six evolved strains (Table 1), revealing 12 to 28 mutations per strain. In strains sharing a common lineage, up to 10 of the mutations were present in both strains. However regardless of lineage, we found recurrent mutations in two loci relevant to acetic acid and ethanol production; in subunits of the *hfs* hydrogenase gene operon and in *adhE*, the bifunctional acetaldehyde alcohol dehydrogenase (Table 2). We thought either of these mutant loci might contribute to the acetate minus phenomenon; entire deletion of the *hfs* hydrogenase gene operon has previously been shown to reduce acetic acid production [18], while mutations in *adhE* could result in a higher affinity for acetyl-CoA or NAD(P)H, effectively out-competing acetic acid or hydrogen formation, respectively. To test these possibilities, we introduced the *hfs* and *adhE* mutations that had occurred in evolved strain M0863 into the wildtype background via homologous recombination with selection based on insertion of a kanamycin resistance marker 3′ to the genomic region of interest. Depending on the exact site of recombination crossover, the mutations were not always carried along with the marker. Strains with the desired mutation were identified by DNA sequencing, and strains without mutations were also evaluated to determine effects that introduction of the resistance marker may have introduced. Results are shown in Table 3. While the mutant *adhE* gene had an impact on end product ratios, only the strain carrying the *hfs* spontaneous mutations produced significantly less acetic acid, with a combined ethanol and lactate to acetate ratio 10-fold higher than wildtype. Introduction of the M0863-acquired *hfs* mutations did not appear detrimental to growth rate, which was in contrast to a complete deletion of the *hfs* operon, which decreased growth rate and the amount of consumed carbohydrate [18].

**Table 1:** **Strains used in this study**

**Strain**	**Description**	**Source**
M0010	*T. saccharolyticum* JW/SL-YS485 DSM 8691	DSMZ
M0355	*T. saccharolyticum* Δ*pta* Δ*ack* Δ*ldh*	(16)
M1291	*T. saccharolyticum* Δ*pta* Δ*ack* Δ*ldh Cth ureABCDEFG*	This study
M1442	*T. saccharolyticum* Δ*pta* Δ*ack* Δ*ldh Cth ureABCDEFG*	This study
M1667	*T. saccharolyticum* Δ*pta* Δ*ack* Δ*ldh::Tsa pta ack kanR Cth ureABCDEFG*	This study
M2030	*T. saccharolyticum* Δ*pta* Δ*ack* Δ*ldh::Tsa pta ack Cac thl Tme thl Tte thl Cac ctfAB Tme ctfAB Cac adc Bam adc kanR Cth ureABCDEFG*	This study
M2202	T. saccharolyticum adhE*::kanR	This study
M2203	*T. saccharolyticum adhE::kanR*	This study
M2204	T. saccharolyticum hfs*::kanR	This study
M2205	*T. saccharolyticum hfs::kanR*	This study
M2212	*T. saccharolyticum* Δ*pta* Δ*ack* Δ*ldh::Tsa pta ack Tte thl Tme ctfAB Bam adc kanR Cth ureABCDEFG*	This study
Top10	*E. coli* cloning strain	Invitrogen, Madison, WI
FY2	*S. cerevisiae* cloning strain	(37)
	Re-sequenced strains with improved growth rate	
M0694	*T. saccharolyticum* Δ*pta* Δ*ack* Δ*ldh* continuous cultured adapted	This study
M0699	*T. saccharolyticum* Δ*pta* Δ*ack* Δ*ldh* continuous cultured adapted	This study
M0700	*T. saccharolyticum* Δ*pta* Δ*ack* Δ*ldh* continuous cultured adapted	This study
M0731	*T. saccharolyticum* Δ*pta* Δ*ack* Δ*ldh* mutation and serial transfer	This study
M0734	*T. saccharolyticum* Δ*pta* Δ*ack* Δ*ldh* mutation and serial transfer	This study
M0863	*T. saccharolyticum* Δ*pta* Δ*ack* Δ*ldh* mutation and serial transfer	This study

DSMZ, Deutsche Sammlung von Mikroorganismen und Zellkulturen.

**Table 2:** **Mutations identified by re-sequencing**

**Gene**	**Locus tag**	**ORF nt**	**DNA mutation**	**Coding change**	**Occurrence in strain**
*adhE*	Tsac_416	1630	G → A	G → D	M0734, M0863
*adhE*	Tsac_416	1804	C → T	S → L	M0699
*adhE*	Tsac_416	1808	A → G	E → G	M0694, M0700
*hfsA*	Tsac_1550	159	A → -	Frameshift	M0700
*hfsB*	Tsac_1551	434	A → -	Frameshift	M0734, M0863
*hfsB*	Tsac_1551	652	A → -	Frameshift	M0731
*hfsC*	Tsac_1552	255	A → -	Frameshift	M0699
*hfsD*	Tsac_1553	193	G → A	A → T	M0699
*hfsD*	Tsac_1553	321	A → T	R → S	M0863
*hfsD*	Tsac_1553	787	G → A	E → K	M0734

**Table 3:** **Re-introduction of spontaneous** ***adhE*** **and** ***hfs*** **hydrogenase mutations that occurred during growth adaptation of the** Δ***ldh*** **,** Δ***pta*** Δ***ack*** **ethanologen strain M0863 into wildtype (WT)** ***T. saccharolyticum***

		**Units in mM**				
**Strain**	**Genotype**	**Cellobiose consumed**	**Lactate**	**Acetate**	**Ethanol**	**Carbon recovery (%)**
M0010	WT	28.2 ± 0.6	13.8 ± 0.5	34.7 ± 0.7	63.5 ± 2.3	99
M2202	adhE*::kan ^*R*^	30.2 ± 0.0	2.4 ± 0.1	42.3 ± 0.3	76.5 ± 0.6	100
M2203	*adhE::kan* ^*R*^	27.7 ± 2.6	1.4 ± 0.2	29.0 ± 1.6	78.4 ± 8.6	98
M2204	hfs*::kan ^*R*^	29.9 ± 0.1	20.3 ± 0.5	4.2 ± 0.1	91.7 ± 0.6	97
M2205	*hfs::kan* ^*R*^	28.1 ± 0.7	9.9 ± 2.2	29.8 ± 1.1	67.9 ± 2.7	96

*mutations found in evolved strain M0863 introduced by homologous recombination. Data are average of four replicates, anaerobic TSC7 medium, 55°C, 24-h fermentation of 30.4 ± 0.7 mM cellobiose. Mutations were created via homologous recombination of a kanamycin resistance marker downstream of the *hfs* operon that contained mutations in the upstream flanking region. M2202 - kan^R^ inserted 3′ of the *adhE* gene, with the M0863 mutant *adhE* sequence. M2203 - kan^R^ inserted 3′ of the *adhE* gene, with the WT *adhE* sequence. M2204 - kan^R^ inserted 3′ of the *hfs* gene operon, with the M0863 mutant *hfs* sequence. M2205 - kan^R^ inserted 3′ of the *hfs* gene operon, with the WT *hfs* sequence.

### Acetic acid and acetyl-CoA freely interconvert in evolved ethanologen strains harboring *ack* and *pta*

**Figure 3:**
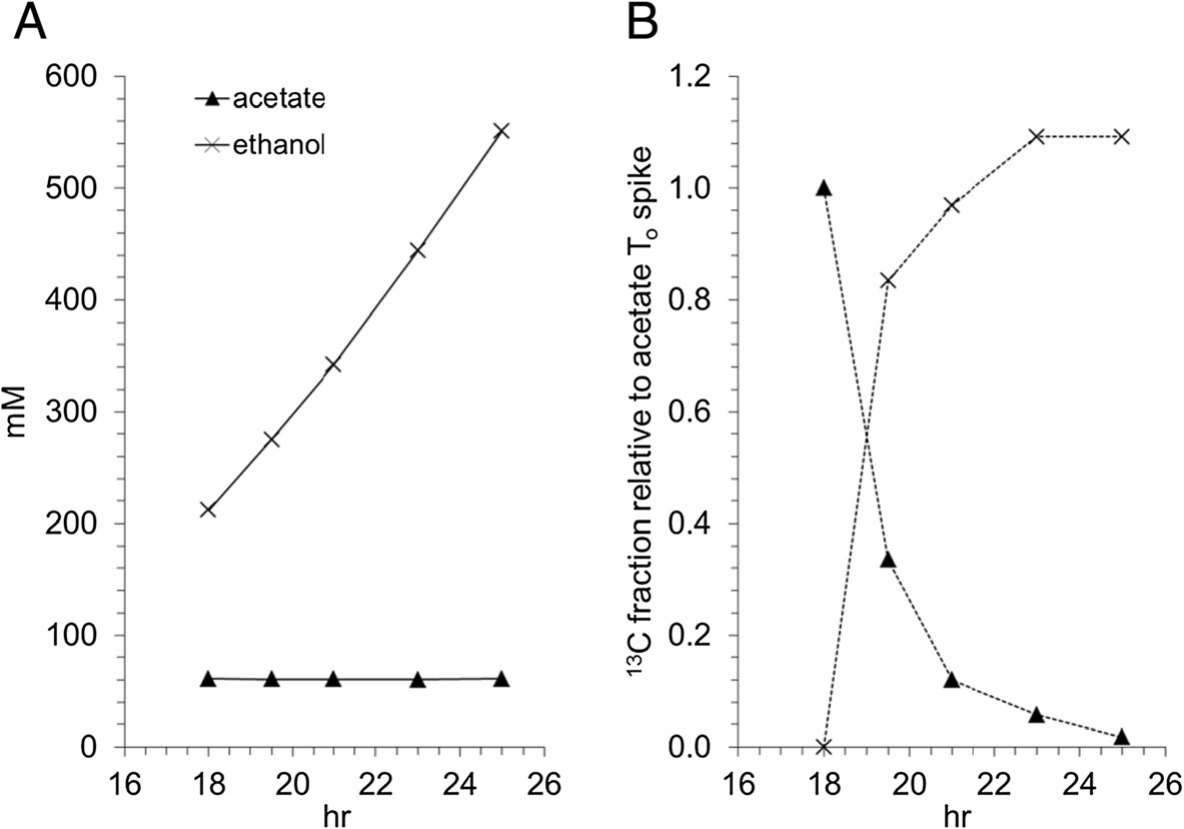
Fate of ^13^C_2_-labeled acetate during strain M1667 carbohydrate fermentation. Strain M1667 was grown in batch fermentation in a 3:2 weight mixture of maltodextrin and cellobiose at a concentration of 900 mM glucose equivalents and 45 mM acetic acid at an initial pH of 6.0. At 18 hours, the culture was spiked with 15 mM sodium acetate-^13^C_2_. Bulk metabolite measurements **(A)** and ^13^C label **(B)** were monitored for extracellular acetic acid (filled triangles) and ethanol (crosses). No appreciable label was found in metabolites other than acetic acid and ethanol. Data is from one representative experiment.

Figure 3 shows ^13^C_2-_labeled acetate spiked at 18 hours in a batch carbohydrate fermentation with strain M1667 (an evolved ethanologen from the M0863 lineage with re-introduced *ack* and *pta* genes) in the presence of 60 mM externally added acetic acid. Based on nuclear magnetic resonance (NMR) spectra, the ^13^C_2_ label is almost completely transferred from acetic acid to ethanol within 7 hours, despite minimal change in the bulk acetic acid concentration. This data indicates a dilution of the ^13^C_2_ acetate pool by approximately 50% in an hour and a corresponding increase in ^13^C_2_ ethanol. No other metabolite showed enrichment of ^13^C beyond that expected from natural abundance indicating that the acetate was metabolized intact. In similar experiments with strain M1291, the direct parent strain of M1667 without *ack* and *pta*, there was no evidence of ^13^C label transfer from acetic acid for up to 48 hours after spike addition.

The reaction acetic acid + ATP + CoA ↔ acetyl-CoA + ADP + P_i_ is near thermodynamic equilibrium (+2 kJ/mol at pH 7) and is used by microorganisms for both acetate production and consumption in various environmental conditions [19-22]. Although the bulk concentration of acetic acid remained constant in evolved ethanologen strains containing *pta* and *ack*, we hypothesize that labeled acetate is reversibly converted into labeled acetyl-CoA, a fraction of which is then irreversibly converted into labeled ethanol. The intracellular acetyl-CoA pool is also fed by unlabeled carbohydrate metabolism and, assuming that ^13^C label is not differentially recognized, can be equally used to produce acetate or ethanol. Carbohydrate-derived acetyl-CoA then acts to dilute the labeled acetate/acetyl-CoA pool and allows the creation of labeled ethanol. Due to stoichiometric coupling with hydrogen formation, net acetate formation is arrested via the evolutionarily acquired *hfs* mutations while the capacity to convert acetate to acetyl-CoA is retained.

### Construction of a synthetic pathway for the conversion of acetic acid to acetone

**Figure 4:**
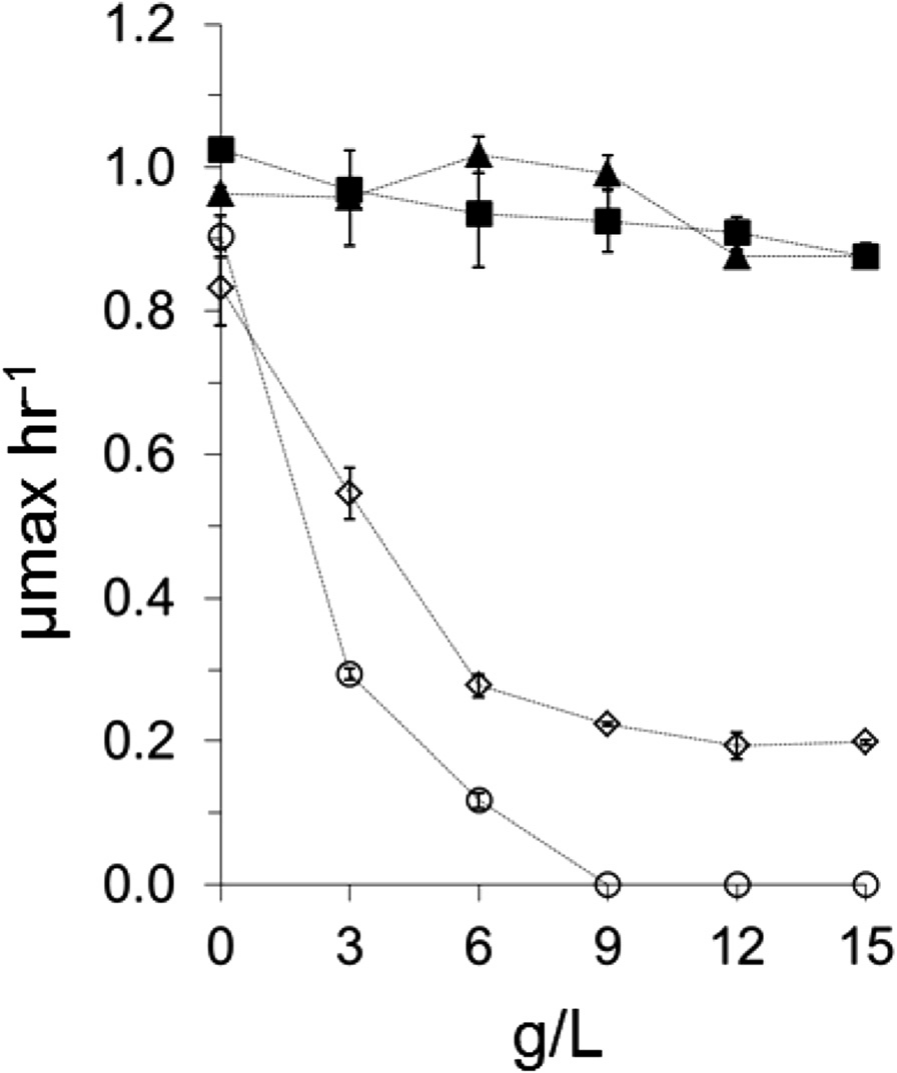
Maximum growth rate of *T. saccharolyticum* in the presence of exogenously added acetic acid or acetone at pH 5.0 and 5.5 in TSC7 medium with 10 g/L cellobiose at 55°C. Open circles - acetic acid pH 5.0, open diamonds - acetic acid pH 5.5, filled squares - acetone pH 5.0, filled triangles - acetone pH 5.5. Data represent the mean of triplicate cultures +/− one standard deviation.

Exogenously added acetone is substantially less inhibitory than acetic acid to *T. saccharolyticum* in the pH range of 5.0 to 5.5 relevant to industrial fermentation (Figure 4). Additionally, conversion of two molecules of acetic acid results in one molecule of acetone, further lowering the potential for acetone inhibition. Solventogenic Clostridia such as *C. acetobutylicum* have native biochemical pathways to convert acetic acid into acetone, although they are intricately coupled to carbohydrate metabolism. We began by importing the *C. acetobutylicum* acetone pathway, inclusive of thiolase, acetate:acetoacetyl-CoA-transferase, and acetoacetate decarboxylase (*thl ctfAB adc*) as well as the *T. saccharolyticum* acetate kinase and phosphotransacetylase into the *T. saccharolyticum* expression plasmid pMU1299 that integrates at the *ldh* locus. Despite conferring acetone production in *E. coli* (Table 4), we could find no evidence of acetone formation upon integration of the plasmid in *T. saccharolyticum*. While RT-PCR experiments (not shown) indicated transcriptional activity of the acetone pathway genes, a likely issue was temperature incompatibility of the donor and host organism; *C. acetobutylicum* has grown optimally between 30°C and 37°C, while *T. saccharolyticum*’s growth rate drops precipitously below 48°C, the lowest temperature where we assayed for acetone formation.

**Table 4:** **Acetone production in** ***E.coli***

	**Glucose**	**Lactic**	**Acetic**	**Ethanol**	**Acetone**
Media	24.5	0.0	4.2	0.0	0.0
TOP10 + pMU1299	16.1	5.9	3.1	0.5	1.8
TOP10 + pACYC177	19.5	3.6	5.1	0.4	0.0

Fermentation end point at 170 hours, in LB media supplemented with 25 g/L glucose, 4 g/L acetic acid, and 50 μg/mL kanamycin sulfate. Microaerobic conditions were utilized by inoculation into sealed 125 mL pressure bottles with atmospheric pressure air. Incubation was performed without agitation at 30°C, with a 1/10^th^ v/v inoculation from aerobically grown overnight LB media supplemented with kanamycin.

**Figure 5:**
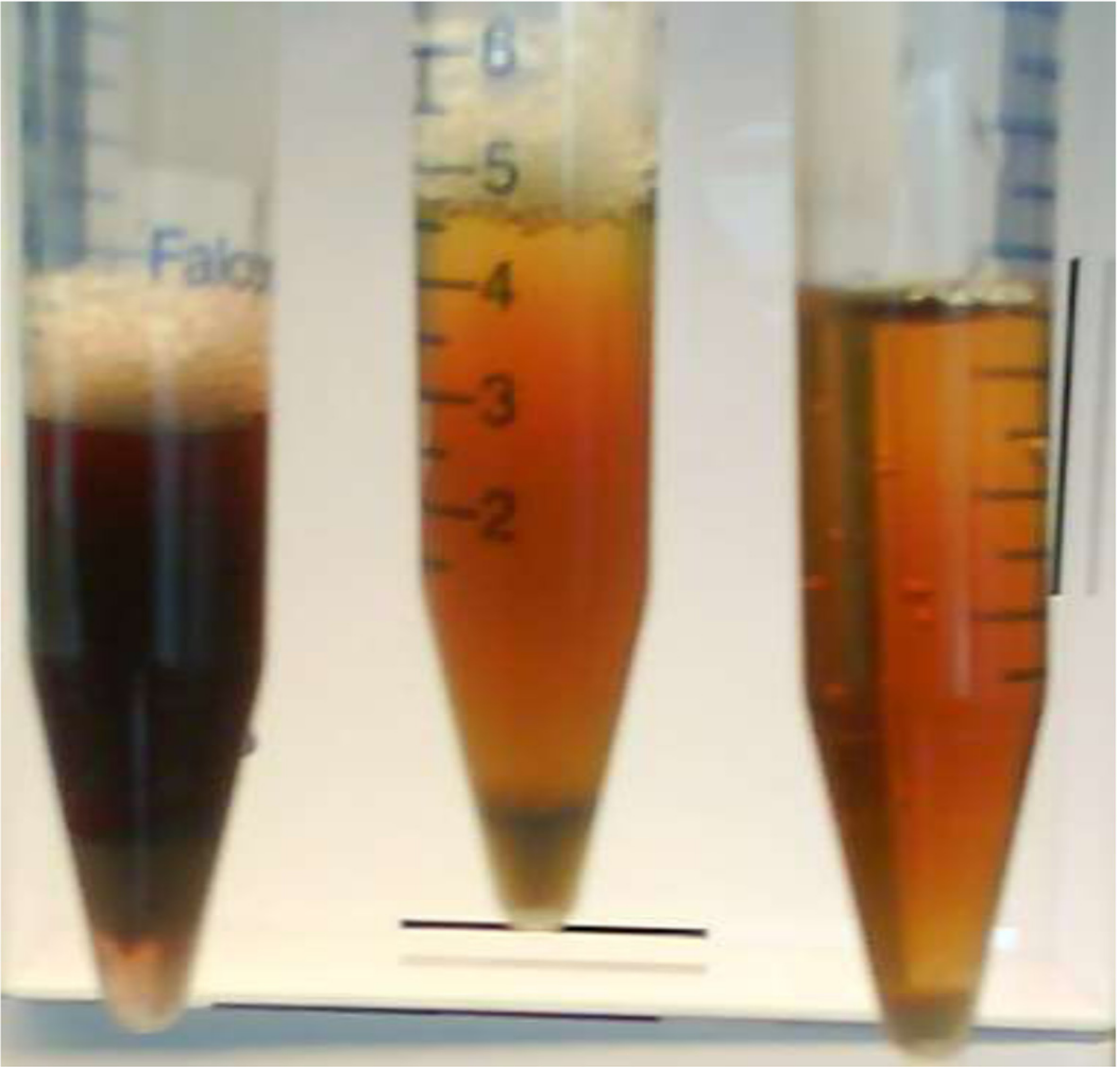
Rothera’s test (1) for acetone and acetoacetic acid formation performed on spent fermentation medium, initially containing 50 g/L cellobiose and 5 g/L acetic acid. From left to right, M2030, M1675 + pMU131 (the immediate parent strain of M2030), and uninoculated medium. Acetone and acetoacetic acid are characterized by a purple/red coloremetric reaction with sodium nitroprusside in the presence of saturating ammonia. To perform the test, 4-mL samples were saturated with ammonium sulfate, and 5% sodium nitroprusside was added followed by an overlay of 1 mL 25% ammonium hydroxide. Photograph was taken 18 hours after addition of sodium nitroprusside.

We next looked for thermophilic acetone producers but could not find a native acetone producer with an optimal growth temperature higher than 43°C [23]. We could, however, find homologs to each of the three enzymes in the acetone pathway residing in the sequenced genomes of thermophilic bacteria (Table 5). None of these thermophiles were reported to produce acetone, and the entire pathway was not present in a single organism. To explore the ability of these genes to produce acetone, we opted to progressively incorporate expression vectors harboring thermophilic acetone pathway homologs into *T. saccharolyticum* and assay the resulting strains for acetone production. Qualitative evidence of ketone body formation was first identified in strain M2030 (Figure 5), which contained, in addition to the *T. saccharolyticum ack* and *pta* genes, three *thl* genes, two *ctfAB* gene operons, and two *adc* genes. We then constructed strains with minimal sets of acetone pathway genes while continuing to assay for acetone formation; the most efficient acetone producer, M2212, was created by expression of a putative thiolase from *Thermoanaerobacterium thermosaccharolyticum* DSM 571, a putative CoA-transferase from *Thermosipho melanesiensis* DSM 12029, and a putative acetoacetate decarboxylase from *Bacillus amyloliquefaciens* FZB42. The *T. thermosaccharolyticum thl* is part of a gene operon with several homologs involved in butanol production, and the organism has been reported to produce butanol [24]. *T. melanesiensis ctfAB* is adjacent to a thiolase in its native genetic organization, which could potentially be involved in acetoacetate degradation. The *B. amyloliquefaciens adc* appears to be independently transcribed; however, the native function of either enzyme is not obvious based on genetic organization and the known physiology of these organisms.

**Table 5:** **Genes tested for acetone production in** ***T.saccharolyticum***

**Source strain**	**T**_**opt**_ **(°C)**	**Gene**	**Genbank protein**
*Thermoanaerobacterium saccharolyticum* DSM 8691	60	*pta*	ACA51668
*Thermoanaerobacterium saccharolyticum* DSM 8691	60	*ack*	ACA51669
*Clostridium acetobutylicum* ATCC 824	35	*thl*	NP_349476
*Thermosipho melanesiensis* DSM 12029	70	*thl*	YP_001306374
*Kosmotoga olearia* DSM 21960	65	*thl*	YP_002940320
*Thermoanaerobacterium thermosaccharolyticum* DSM 571	60	*thl*	YP_003852249
*Thermoanaerobacterium thermosaccharolyticum* DSM 571	60	*actA*	CAA93155
*Clostridium acetobutylicum* ATCC 824	35	*ctfA*	NP_149326
*Thermosipho melanesiensis* DSM 12029	70	*ctfA*	YP_001306376
*Kosmotoga olearia* DSM 21960	65	*ctfA*	YP_002940319
*Clostridium acetobutylicum* ATCC 824	35	*ctfB*	NP_149327
*Thermosipho melanesiensis* DSM 12029	70	*ctfB*	YP_001306375
*Kosmotoga olearia* DSM 21960	65	*ctfB*	YP_002940318
*Clostridium acetobutylicum* ATCC 824	35	*adc*	NP_149328
*Acidothermus cellulolyticus* B11 ATCC 43068	55	*adc*	YP_872855
*Bacillus amyloliquefaciens* FZB42 BGSC 10A6	50	*adc*	YP_001422565

T_opt_ is the optimal temperature for growth of the source strain.

Strain M2212 was also assayed for the *in vitro* ability to catalyze conversions along the pathway of acetyl-CoA to acetone. Table 6 shows data collected from cell-free extract measurements of thiolase, CoA-transferase, and acetoacetate decarboxylase from strain M2212 and M1442, the direct parent ethanologen. While activities for these enzymes were at or below the limit of detection with strain M1442, detectable levels were found for each step with strain M2212.

**Table 6:** **Enzymatic activities in cell extracts**

	**μmol min** ^**−1**^ **mg** ^**−1**^ **protein**					
	**Thiolase**	**SD**	**CoA-transferase**	**SD**	**Acetoacetate decarboxylase**	**SD**
M1442	0.06	0.13	0.02	0.02	0.17	0.23
M2212	10.21	0.55	3.57	0.09	1.66	0.26

Data represent the mean of triplicate determinations +/− one standard deviation. SD, standard deviation.

### Expression of the synthetic acetone pathway improves ethanol productivity and titer in the presence of acetic acid

**Figure 6:**
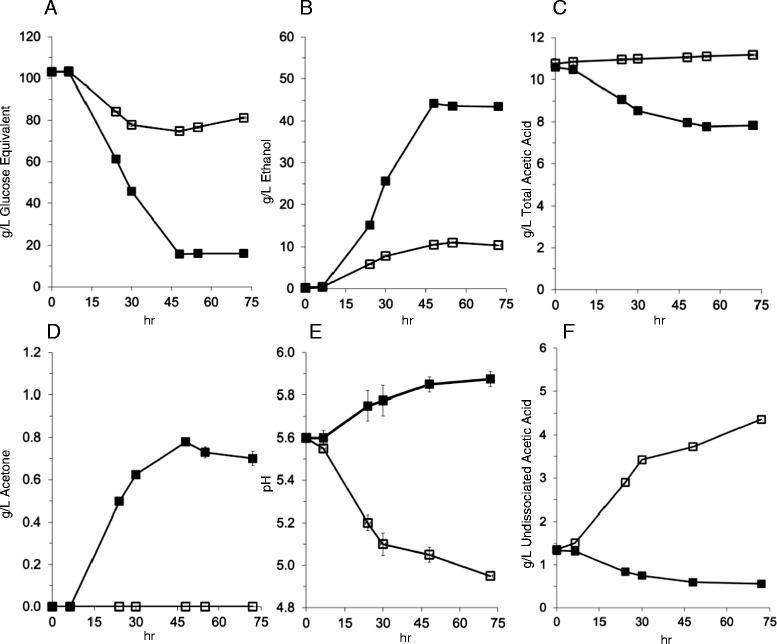
Fermentation of maltodextrin, plotted as glucose equivalents, in the presence of 10 g/L acetic acid in TSC7 medium at 55°C. **(A-F)** show the levels of different substrates, products or pH, as indicated by the vertical axis label. Filled squares - M2212, containing the acetic acid to acetone pathway. Open squares - parental ethanologen strain M1442. Strain M1442 rapidly encounters inhibition as the undissociated acetic acid concentration rises, while M2212 decreases the undissociated acetic acid to below 0.6 g/L **(F)**. Data represent the mean of triplicate cultures +/− one standard deviation.

Strain M2212 and strain M1442 were grown in batch fermentation in the presence of acetic acid (Figure 6). M2212 reduced the overall acetic acid concentration by 2.8 g/L, which in combination with an increase in the pH, reduced the undissociated acetic acid concentration from an initial 1.3 g/L to below 0.6 g/L. 0.78 g/L acetone was produced, a value lower than that expected from the theoretical 0.5 g/g conversion of acetic acid to acetone. However, this measurement may also reflect some acetone evaporation during fermentation gas release, as the boiling point of acetone, 54°C, is slightly below the 55°C fermentation temperature. In contrast, fermentation with strain M1442 resulted in a small 0.4 g/L increase in acetic acid, while the undissociated acetic acid concentration rose to over 4.3 g/L as a result of declining pH during the fermentation. From Figure 4, a 50% decrease in μmax occurs at an undissociated acetic acid concentration of 0.61 to 0.81 g/L. The decline in pH with strain M1442 is likely a result of assimilation of ammonium sulfate and carbonic acid generated during production of carbon dioxide. The conversion of acetic acid to acetone completely reversed this pH trend in strain M2212.

Strain M2212 has favorable ethanol fermentation metrics as well; compared to M1442, the final ethanol titer was 33.1 g/L higher, an increase of more than 300%. Volumetric productivity at 48 hours rose from 0.22 g/L hr^−1^ to 0.92 g/L hr^−1^, also an increase of over 300%. Final metabolic yields, as a measure of gram ethanol produced per gram glucose equivalent consumed, were 0.48 g/g for M1442 and 0.50 g/g for M2212.

## Discussion

The observation that re-introduction of *ack* and *pta* in evolved ethanologen strains resulted only in minute amounts of acetic acid production was surprising; the genes had initially been removed to eliminate acetic acid production, which can account for up to 30% of total organic end products on a molar basis in wildtype cells [17,25]. Predicting that evolutionary selection had imparted mutations resulting in this phenotype, we examined the genome sequences of several strains descended from strain M0355 that had been serially or continuously cultured for several generations. We identified two mutational hotspots, in the *hfs* hydrogenase gene operon and the *adhE* bifunctional acetaldehyde/alcohol dehydrogenase, that could be rationally predicted to impact acetic acid production. Introducing these mutations to the wildtype strain indicated that the *hfs* mutation is responsible for a sharp decrease in acetic acid production.

An entire deletion of the *hfs* operon has previously been shown to reduce acetic acid production in wildtype and Δ*ldh T. saccharolyticum* strains [18]. However, it also results in strains with poor fermentation kinetics, which are not readily improved after several serial transfers. Deletion of the entire *hfs* operon in evolved Δ*ldh* Δ*ack* Δ*pta* ethanologens also resulted in strains with poor fermentation kinetics, suggesting that the point mutations acquired through evolution have a more nuanced effect than an entire loss of function mutation. Any fitness advantage the *hfs* mutations impart to ethanologen strains is unknown, although it is tempting to speculate that cellular redox regulation may play a role. Of six evolved strains, five acquired frameshift mutations in the genes *hfsA*, *hfsB*, or *hfsC*, encoding a putative ferredoxin, a hydrogenase and [PAS] sensory domain fusion and a histidine kinase, respectively. Identification and classification of [FeFe] hydrogenases with possible sensory functions is an area of current investigation [26-28]. These frameshift mutations may be beneficial by loss of function of the affected gene but may also have beneficial effects through polar down-regulation of the gene *hfsD* downstream. This gene, encoding a putative monomeric group B [FeFe] hydrogenase [28], only acquired point mutations, resulting in amino acid changes in three of six strains. A small amount of hydrogen production can be predicted to be important for ethanologen strains to maintain redox balance with cell mass and associated carbon dioxide generation from carbohydrates, in a role parallel to that of glycerol formation in *S. cerevisiae* [29-31].

The *adhE* mutations, although not further investigated here, may be involved in co-factor specificity and increased ethanol tolerance, as previously reported for *C. thermocellum* [32]. Another evolved *T. saccharolyticum* ethanologen, strain ALK2, was shown to have an increase in nicotinamide adenine dinucleotide phosphate (NADPH) specificity and a decrease in NADH specificity for whole cell extract alcohol dehydrogenase activity [17].

The combination of *hfs* gene mutations and *ack* and *pta* re-introduction allows for inter-conversion of acetic acid and acetyl-CoA independent of central metabolism. To detoxify acetic acid with concurrent high yield fermentation of carbohydrates to ethanol, the solvent acetone was an attractive end product of acetic acid metabolism. It is less inhibitory than acetic acid, can be easily recovered through distillation, and can be sold as a commodity chemical. The native solventogenic clostridial pathway takes one acetyl-CoA and one acetic acid and converts them to one CoA, one CO_2_, and one acetone. This, combined with action of phosphotransacetylase and acetate kinase, allows the redox neutral conversion of two acetic acid and one ATP to one acetone, one CO_2_, one H_2_O, and one ADP + P_i_. The standard Gibbs free energy change for this reaction is −0.8 kJ/rxn, a fairly small driving force. However, if acetic acid accumulates intracellularly due to a transmembrane pH gradient, the overall reaction will become increasingly thermodynamically favorable. Assuming acetic acid is at intracellular and extracellular equilibrium, an extracellular change of 1.0 pH unit more acidic results in a ΔG’ change of −12.4 kJ/rxn. Thus, the pathway becomes more thermodynamically feasible as acetic acid becomes more inhibitory.

The inability to produce acetone in *T. saccharolyticum* with heterologously expressed enzymes from *C. acetobutylicum* and the lack of natively thermophilic acetone producers led us to test homologs of thiolase, CoA-transferase, and acetoacetate decarboxylase from thermophiles that presumably participate in other metabolic pathways. The final pathway reported here, utilizing genes from *T. saccharolyticum*, *T. thermosaccharolyticum*, *T. melanesiensis*, and *B. amyloliquefaciens*, is assembled entirely from organisms that do not produce acetone from acetic acid.

Despite being carbon and electron independent, production of acetone from acetic acid does require chemical energy in the form of ATP, which must be supplied from central catabolism. Based on a stoichiometric evaluation of the fermentation presented in Figure 6, 5% of the total ATP produced by carbohydrate catabolism is used for acetic acid conversion to acetone during the maximum rate of acetone formation. The rapid and complete conversion of ^13^C-labeled acetate to ethanol indicates there is sufficient capacity for acetate entry into the cell. Further conversion could be limited by thermodynamic feasibility, as the majority of the residual acetic acid is in the dissociated species when the rate of acetone production decreases.

From economic and process engineering perspectives, biological conversion of acetic acid to acetone provides several distinct advantages over other detoxification methods. In the case of lignocellulose to ethanol conversion, it is likely to increase ethanol productivity and titer, while maintaining the carbohydrate to ethanol yield essential for efficient biofuel production. Acetone is readily recovered by distillation equipment already installed for ethanol recovery, and it has a selling price around $1.15 per kg [33] with a North American market size of 1.6 MM metric tons per year [34]. The conversion of acetic acid to a solvent reduces the demand for pH neutralization during fermentation and lowers the chemical or biological oxygen demand for wastewater treatment during water recycle, as acetic acid composes half or more of soluble, unrecovered organic material in a typical lignocellulose process [35]. Finally, unlike chemical or physical methods to remove acetic acid, conversion to acetone does not require significant additional capital or operating expenditures, as new unit operations are not required beyond separation of acetone from ethanol. With these factors taken together, this technology has the potential to lower costs and improve the feasibility of large scale, anaerobic production of biofuels and biochemicals from acetate containing feedstocks.

## Conclusions

Spontaneous mutations acquired in the *hfs* hydrogenase gene cluster prevent net acetate production in strains in which the *pta* and *ack* genes are re-introduced. Surprisingly, uptake and incorporation of exogenous acetate occurs in those strains even when overall acetate levels remain constant, as shown by ^13^C-labeling. Genes from *C. acetobutylicum* did not confer a functional acetone production pathway, but genes assembled from a variety of thermophilic species did. The best performing strain converted exogenous acetate to acetone, while markedly improving ethanol production. These findings improve our understanding of acetate and ethanol metabolism and demonstrate a novel strategy for converting a potential inhibitor into a valuable co-product.

## Methods

### Strains and DNA vectors

Strains used in this study are listed in Table 1, and plasmids and primers are given in Additional file 1. Plasmids used to transform *T. saccharolyticum* were constructed with *S. cerevisiae* gap repair cloning techniques and amplified in *E. coli* [36]. *T. saccharolyticum* was transformed using a natural competence based system [37], and transformants were selected by resistance to the antibiotic kanamycin. Chromosomal integrants were evaluated by colony PCR with primers which hybridized to regions external and internal to the integration region.

### Fermentation conditions

*T. saccharolyticum* was primarily grown in modified TSC7 medium, containing per liter 1.0 g sodium citrate tribasic dihydrate, 4.0 g (NH_4_)_2_SO_4_, 0.2 g FeSO_4_•7H_2_O, 2.0 g MgSO_4_•7H_2_O, 1.0 g KH_2_PO_4_, 0.2 g CaCl_2_•2H_2_O, 0.12 g L-methionine, 0.5 g L-cystiene HCl, and 8.5 g yeast extract. For C^13^ acetate labeling experiments, TSC4 base medium was used, with the following differences from TSC7: 5 g urea replacing (NH_4_)_2_SO_4_, 0.5 g MgSO_4_•7H_2_O, 0.5 g KH_2_PO_4_, and 2 g sodium citrate tribasic dihydrate. Carbohydrates and acetic acid were added as indicated in the text and figure legends. Chemicals, including ^13^C_2_ acetate, were acquired from Sigma-Aldrich (St Louis, MO, USA). Fermentations were begun by addition of 10% *v*/*v* from an exponentially growing pre-culture and were performed in sealed pressure vessels (120-mL bottles with a 20-mL working volume for high carbohydrate concentrations and 20-mL tubes with a 5-mL working volume for low carbohydrate concentrations. Vessels were purged with an anaerobic gas mixture containing 95% N_2_ and 5% CO_2_ and vented to atmospheric pressure at each sampling. Fermentations were performed at 55°C and shaking at 150 to 200 rpm.

### Analytical methods

Fermentation metabolite concentrations were determined by high-performance liquid chromatography (HPLC) using an Aminex HPX-87H column (Bio-Rad Laboratories, Hercules, CA, USA) with a refractive index detector. Acetone was additionally identified with a UV detector at 260 nm. Proton-decoupled one-dimensional ^13^C spectra were obtained from fermentation samples mixed with D_2_O (20%) with a Varian Inova 500MHz NMR spectrometer (Bloomington, IN, USA) operating at 125 MHz using the WALTZ-16 sequence for decoupling. The sweep width was 18 kHz, and acquisition time was 1.5 s. A pulse angle of 80° and 0.8 s delay were used. Data (65,000 pts) was Fourier transformed with a line broadening of 0.5 Hz.

Ketone body formation was qualitatively assayed via the Rothera method, as described in Figure 5. Maltodextrin was hydrolyzed with *A. niger* amyloglucosidase according to the starch assay kit protocol (Sigma-Aldrich, St. Louis, MO, USA) prior to detection of glucose by HPLC. Undissociated acetic acid levels were calculated from acetate and pH measurements.

### Genome re-sequencing

Descendants of the ethanologen strain M0355 evolved for improved growth rate and ethanol production via continuous culturing and chemical mutagenesis were sequenced at the National Center for Genome Resources on the Illumina platform, with each strain generating 5 to 8 million 36 bp reads. The reads were assembled onto a draft genome of wildtype *T. saccharolyticum* reference sequence using SeqMan NGen from DNAStar (Madison, WI, USA). SNPs, and other small mutations were manually verified by examination of the sequence assemblies and distinguished from errors in the draft genome sequence, evident as sequence differences in all strains.

### Enzymatic assays

Cell-free extracts were prepared from cultures grown in 200 mL TSC7 medium with 10 g/L cellobiose at 55°C to an optical density of 1.0. Cells were chilled to 4°C, harvested aerobically by centrifugation at 8,000×*g* for 5 min, washed twice with 50-mL cold water, and pelleted to approximately 250-uL wet volume. Cell lysis was performed by addition of B-PER bacterial cell lysis reagent (Thermo Scientific, Rockford, IL, USA) with 0.1 mg lysozyme, 2.5 U DNAse I, and 1 mM phenylmethanesulfonylfluoride per mL of B-PER reagent. Cell extracts used for acetoacetate decarboxylase assays additionally contained 50 mM dithiothreitol. After cell re-suspension and a 30-min room temperature incubation, cell debris was removed by centrifugation at 15,000×*g* for 10 min. Protein concentration was determined via the Bradford method with Bio-Rad Protein Assay Reagent (Bio-Rad, Hercules, CA, USA). All chemicals and enzymes were obtained from Sigma-Aldrich, and reported data are the average of three replicate assays.

Thiolase was assayed in the direction of acetoacetyl-CoA cleavage, which was monitored at 303 nm with an extinction coefficient of 14.00 mM^−1^ cm^−1^ at room temperature in a Spectramax M2 plate reader (Molecular Devices, Sunnyvale, CA, USA). The assay contained, in a final volume of 200 uL, 100 mM Tris-HCl (pH 8.0), 1 mM DTT, 10 mM MgCl_2_, 0.05 mM acetoacetyl-CoA, 0.2 mM CoA and 4 μg cell-free extract. Addition of CoA started the reaction, and activity depended on both the presence of acetoacetyl-CoA and cell-free extract.

CoA-transferase was assayed in the direction of acetate and acetoacetyl-CoA conversion to acetoacetate and acetyl-CoA. To measure activity, acetyl-CoA was subsequently converted to citrate and CoA via citrate synthase, and CoA formation was monitored with 5,5′-dithiobis-(2-nitrobenzoic acid) (DTNB) at 412 nm with an extinction coefficient of 14.15 mM^−1^ cm^−1^ at room temperature in a Spectramax M2 plate reader. The assay contained, in a final volume of 200 uL, 67 mM Tris-HCl (pH 8.0), 10 mM potassium acetate, 1 mM oxaloacetate, 0.1 mM DTNB, 2 U porcine heart citrate synthase, 0.2 mM acetoacetyl-CoA, 0.2 mM CoA, and 4 μg cell-free extract. Addition of acetoacetyl-CoA started the reaction, and activity depended on the presence of acetoacetyl-CoA, acetate, and cell-free extract. Control assays without added cell extract were used to subtract baseline signal.

Acetoacetate decarboxylase was assayed in the direction of acetone formation via HPLC with a Fast Acid Analysis column (Bio-Rad, Hercules, CA, USA) with refractive index and UV detection at 260 nm. The assay contained, in a final volume of 1.8 mL, 100 mM KPO_4_ buffer at pH 6.1, 25 mM lithium acetoacetate and 180 μg cell-free extract. The assay began with addition of cell-free extract, and samples were taken at 70 min and frozen at −80°C until acidification to pH 2 and HPLC analysis. Control assays without added cell extract were used to subtract baseline signal.

## Additional file

Additional file 1:
**Plasmids and primers used in this study.**

## Abbreviations

ADP: adenosine diphosphate
ATP: adenosine triphosphate
CoA: coenzyme A
NADH: nicotinamide adenine dinucleotide
NADPH: nicotinamide adenine dinucleotide phosphate
NMR: nuclear magnetic resonance
RT-PCR: reverse transcriptase-polymerase chain reaction
