# Supplementary material for: Anaerobic detoxification of acetic acid in a thermophilic ethanologen
Source: Biotechnol Biofuels. 2015 May 9;8:75. doi: 10.1186/s13068-015-0257-4 (PMC4898469; doi:10.1186/s13068-015-0257-4)
Supplement: Additional file 1: — Plasmids and primers used in this study. [file 13068_2015_257_MOESM1_ESM.docx]

To: Patrick Hallenbeck	April 9, 2015
Biotechnology for Biofuels Editorial Office <[em@editorialmanager.com](mailto:em@editorialmanager.com)>

Regarding Manuscript BBIO-D-15-00044
Anaerobic detoxification of acetic acid in a thermophilic ethanologen
A Joe Shaw; Bethany B Miller; Stephen R Rogers; William R Kenealy; Alex Meola; Ashwini Bhandiwad; W Ryan Sillers; Indraneel Shikhare; David A. Hogsett; Christopher D. Herring, PhD
Biotechnology for Biofuels

Dear Patrick,

Thank you for the rapid review of out submitted manuscript. We have carefully read through the comments made by the reviewer and Editor, and would like to submit a revised manuscript that addresses them. I apologize that the supplementary figures and tables were not included in the previous submission and I have corrected that problem. I believe that these additional files comprised an important part of our work and have moved all but one into the main body of the manuscript.

In addition, I would like to address the reviewers comments, point by point as follows:

*Reviewer #1: Comments
1.      The supplementary Figures and Tables are missing and thus could not be reviewed.*

We apologize for that oversight, and now provide the missing information with this re-submission. Given that Biotechnology for Biofuels is an online journal, we have moved all but one of the files into the main body of the text. These formerly supplemental files are now Tables 2, 4, & 5, and Figures 2 & 5. The one remaining file consists of primer and plasmid sequences and we feel that it is best to remain as a supplemental file. It too will be provided with this resubmission.

*2.      Page 6, line 7; Table 2. Hydrogen is still produced, but was it measured? Measuring hydrogen would enable you to calculate the electron balance giving clues how the electrons are divided over all byproducts.*

Unfortunately, the equipment required for hydrogen measurement is not available to us, and we are unable to follow the reviewer's recommendation.  Due to the well documented coupling of acetate and hydrogen production from carbohydrates (Thauer RK, et al., Energy conservation in chemotrophic anaerobic bacteria, Bacteriol Rev. 1977 Mar;41(1):100-80), we believe the conclusions reached from the data presented in table 2 remain relevant however. Investigating the biochemical activity of the mutant hydrogenases identified here would be an interesting study, but we believe it is outside the scope of the current work.

*3.      Page 8, last line and further. Strain M2212 is mentioned in the text, but Table 1 refers only to a M2122 strain. Please check.*

This was a typographical error, and the correct strain number should have been M2212 in Table 1. It has been corrected.

*4.      Page 10 line 4. Figure 2 should be Figure 3.*

This has been corrected.

*Editor: Comments
The cited supplementary files were not uploaded with the original submission, thus a complete review was not possible.  Please revise the manuscript in accordance with the reviewers' comments and resubmit the revised version with supplementary files for complete review and a final decision.*

See response to # 1 above.

I hope that these responses adequately address the concerns that were raised. I look forward to your decision and am at your service to provide any additional information needed to proceed with publication.

Sincerely,

Christopher D. Herring, PhD

Dartmouth College

Thayer School of Engineering

14 Engineering Drive

Hanover, NH 03755

Email: [chrisherringfish@gmail.com](mailto:chrisherringfish@gmail.com)

Phone: 603-646-9212
